# Oxidative Stress and Placental Pathogenesis: A Contemporary Overview of Potential Biomarkers and Emerging Therapeutics

**DOI:** 10.3390/ijms252212195

**Published:** 2024-11-13

**Authors:** Ioana Vornic, Victor Buciu, Cristian George Furau, Pusa Nela Gaje, Raluca Amalia Ceausu, Cristina-Stefania Dumitru, Alina Cristina Barb, Dorin Novacescu, Alin Adrian Cumpanas, Silviu Constantin Latcu, Talida Georgiana Cut, Flavia Zara

**Affiliations:** 1 Doctoral School, Department Medicine, “Vasile Goldiș” Western University of Arad, Liviu Rebreanu Street, No. 86, 310414 Arad, Romania; ioana_vornic@yahoo.com; 2 Discipline of Gynecology, Department Medicine, Vasile Goldiş Western University, Liviu Rebreanu Boulevard, No. 86, 310414 Arad, Romania; cristianfurau@gmail.com; 3 Doctoral School, Victor Babes University of Medicine and Pharmacy Timisoara, E. Murgu Square, No. 2, 300041 Timisoara, Romania; silviu.latcu@umft.ro; 4 Department II of Microscopic Morphology, Victor Babes University of Medicine and Pharmacy Timisoara, E. Murgu Square, No. 2, 300041 Timisoara, Romania; gaje.nela@umft.ro (P.N.G.); ra.ceausu@umft.ro (R.A.C.); cristina-stefania.dumitru@umft.ro (C.-S.D.); toma.alina@umft.ro (A.C.B.); novacescu.dorin@umft.ro (D.N.); flavia.zara@umft.ro (F.Z.); 5 Angiogenesis Research Center, Victor Babes University of Medicine and Pharmacy Timisoara, E. Murgu Square, No. 2, 300041 Timisoara, Romania; 6 Department XV, Discipline of Urology, Victor Babes University of Medicine and Pharmacy Timisoara, E. Murgu Square, No. 2, 300041 Timisoara, Romania; cumpanas.alin@umft.ro; 7 Department XIII, Discipline of Infectious Diseases, Victor Babes University of Medicine and Pharmacy Timisoara, E. Murgu Square, No. 2, 300041 Timisoara, Romania; talida.cut@umft.ro; 8 Center for Ethics in Human Genetic Identifications, Victor Babes University of Medicine and Pharmacy Timisoara, E. Murgu Square, No. 2, 300041 Timisoara, Romania

**Keywords:** pregnancy complications, pregnancy loss/miscarriage, pre-eclampsia, maternal–placental–fetal interactions, trophoblast, oxidative stress biomarkers, novel therapeutic approaches, intrauterine fetal growth restriction, placental molecular pathology, DNA damage

## Abstract

Oxidative stress (OS) plays a crucial role in placental pathogenesis and pregnancy-related complications. This review explores OS’s impact on placental development and function, focusing on novel biomarkers for the early detection of at-risk pregnancies and emerging therapeutic strategies. We analyzed recent research on OS in placental pathophysiology, examining its sources, mechanisms, and effects. While trophoblast invasion under low-oxygen conditions and hypoxia-induced OS regulate physiological placental development, excessive OS can lead to complications like miscarriage, preeclampsia, and intrauterine growth restriction. Promising OS biomarkers, including malondialdehyde, 8-isoprostane, and the sFlt-1/PlGF ratio, show potential for the early detection of pregnancy complications. Therapeutic strategies targeting OS, such as mitochondria-targeted antioxidants, Nrf2 activators, and gasotransmitter therapies, demonstrate encouraging preclinical results. However, clinical translation remains challenging. Future research should focus on validating these biomarkers in large-scale studies and developing personalized therapies to modulate placental OS. Emerging approaches like extracellular vesicle-based therapies and nanomedicine warrant further investigation for both diagnostic and therapeutic applications in pregnancy-related complications. Integrating OS biomarkers with other molecular and cellular markers offers improved potential for the early identification of at-risk pregnancies.

## 1. Introduction

Pregnancy represents a unique physiological state characterized by complex adaptations that ensure the survival and growth of the developing fetus. Central to this process is the placenta, an organ that functions as the critical interface between the mother and fetus [1]. The placenta facilitates the exchange of gases, nutrients, and waste products; produces hormones essential for maintaining pregnancy; and modulates immune interactions to prevent fetal rejection [2]. The proper development and function of the placenta are therefore paramount for fetal health and successful pregnancy outcomes.

A key factor influencing placental development and function is the delicate balance between reactive oxygen species (ROS) and reactive nitrogen species (RNS) production and the antioxidant defense mechanisms that neutralize these reactive molecules [3]. ROS and RNS are generated as natural byproducts of cellular metabolism and play essential roles in cell signaling, gene expression, and defense against pathogens [4]. However, an imbalance favoring the accumulation of ROS/RNS leads to oxidative stress (OS), which can damage lipids, proteins, and DNA, disrupt cellular functions, and trigger cell death pathways such as apoptosis and autophagy [5].

In the placenta, OS is a double-edged sword. On one hand, controlled levels of ROS are necessary for normal placental functions, including trophoblast proliferation, differentiation, invasion, and angiogenesis [6]. These processes are crucial during early pregnancy when the placenta develops in a relatively low-oxygen environment [7]. Hypoxia, or low oxygen tension, stimulates the expression of hypoxia-inducible factor-1 (HIF-1), a transcription factor that regulates genes involved in angiogenesis, metabolism, and cell survival [8]. HIF-1 activation under hypoxic conditions promotes the production of vascular endothelial growth factor (VEGF), facilitating the formation of new blood vessels and ensuring adequate oxygen and nutrient supply to the developing fetus [9].

As pregnancy progresses, the placenta undergoes a transition from a hypoxic to a more oxygen-rich environment [10]. This shift is due to the remodeling of maternal spiral arteries by extravillous trophoblasts (EVTs), which replace the endothelial lining and transform the arteries into high-capacity, low-resistance vessels [11]. The ↑ blood flow enhances oxygen delivery to the placenta, but it also exposes placental tissues to higher oxygen levels that can generate ROS through various biochemical pathways, including mitochondrial oxidative phosphorylation and enzymatic reactions involving nicotinamide adenine dinucleotide phosphate (NADPH) oxidases (Nox) and xanthine oxidase (XO) [12].

While the placenta has antioxidant systems to mitigate ROS-induced damage, excessive OS can overwhelm these defenses, leading to pathological conditions [13]. High levels of ROS can impair trophoblast function, inhibit EVT invasion, and disrupt spiral artery remodeling, resulting in reduced placental perfusion and nutrient exchange [14]. These events contribute to pregnancy complications such as preeclampsia (PE), characterized by hypertension and proteinuria, and intrauterine growth restriction (IUGR), where fetal growth is compromised [15].

In PE, inadequate placental perfusion leads to intermittent hypoxia–reoxygenation episodes, exacerbating OS and promoting the release of anti-angiogenic factors like soluble fms-like tyrosine kinase-1 (sFlt-1) and soluble endoglin (sEng) [16]. These factors interfere with normal angiogenic signaling, further impairing placental function and contributing to maternal endothelial dysfunction [17]. Similarly, in IUGR, chronic hypoxia and OS can damage placental vasculature and reduce nutrient and oxygen delivery to the fetus, leading to restricted growth [18].

Moreover, OS influences cellular processes such as autophagy and apoptosis in the placenta [19]. Autophagy, a catabolic process that degrades and recycles cellular components, is activated under stress conditions to promote cell survival by removing damaged organelles and proteins [20]. However, excessive or dysregulated autophagy can lead to autophagic cell death [21]. Apoptosis, or programmed cell death, can be triggered by oxidative damage to cellular components, including mitochondrial dysfunction and deoxyribonucleic acid (DNA) damage [22]. The balance between autophagy and apoptosis is critical for maintaining placental homeostasis; the disruption of this balance by OS can result in placental insufficiency and adverse pregnancy outcomes [23].

Understanding the mechanisms underlying OS in the placenta is essential for developing therapeutic strategies to prevent or mitigate pregnancy complications [24]. Antioxidant therapies, including supplementation with vitamins C and E, have been investigated, but results are mixed, highlighting the need for more targeted approaches [25]. Novel therapies aim to modulate OS by enhancing endogenous antioxidant defenses, promoting angiogenesis, and improving trophoblast function [26]. These include the use of gasotransmitters like nitric oxide (NO), carbon monoxide (CO), and hydrogen sulfide (H_2_S), which have vasodilatory and anti-inflammatory properties [27]. Additionally, gene therapy approaches, targeting angiogenic factors such as VEGF and insulin-like growth factor-1 (IGF-1), hold promise for restoring normal placental development and function [28].

This comprehensive review explores the multifaceted role of OS in placental health and disease. We examine the sources and mechanisms of ROS and RNS generation in the placenta, their impact on trophoblast biology and vascular remodeling, and the cellular responses involving autophagy and apoptosis [29]. We also discuss the antioxidant defense systems inherent to the placenta and consider current and emerging therapeutic strategies aimed at modulating OS to improve pregnancy outcomes [30]. By integrating findings from recent research, we aim to provide a detailed understanding of how OS contributes to placental pathologies and identify potential avenues for intervention.

Through this exploration, we underscore the importance of maintaining oxidative balance in the placenta and highlight the complexities involved in targeting OS therapeutically. Recognizing the dual nature of ROS as both signaling molecules and potential sources of cellular damage emphasizes the need for precision in developing treatments [31]. Future research directions include elucidating the specific pathways by which OS affects placental cells, identifying reliable biomarkers for the early detection of OS-related complications, and tailoring therapies to individual patient profiles for optimal efficacy and safety [32].

In summary, OS is a critical factor in placental development and function, with significant implications for maternal and fetal health. A deeper understanding of its role offers opportunities to improve clinical outcomes in pregnancy-related disorders associated with OS [33].

## 2. Normal Placental Development, Morphology and Function

The placenta is a vital organ in pregnancy, serving as the primary interface for nutrient and gas exchange between the mother and fetus. Proper placental development is crucial for a successful pregnancy and healthy fetal growth. Placental development and function are regulated through a series of complex, coordinated processes that occur throughout gestation, beginning shortly after fertilization.

### 2.1. Placental Organogenesis

The placenta is a transitional organ that begins to form in the early days of pregnancy. Structurally, it is a unique organ because it consists of tissues derived from two distinct organisms: the mother and the fetus. This dual origin allows the placenta to fulfill its primary roles of supporting fetal nutrition, growth, and development while simultaneously protecting the fetus from potentially harmful substances circulating in maternal blood through the materno-fetal barrier. Under normal conditions, the placenta is entirely expelled after birth.

Placental development begins shortly after the implantation of the blastocyst into the uterine wall, typically around 6–7 days post-conception. The blastocyst’s outer layer, known as the trophoblast, differentiates into two main lineages: a villous trophoblast and an EVT. The villous trophoblast forms the placental villi, which are responsible for nutrient and gas exchange, whereas the EVT invades the maternal decidua and remodels spiral arteries, so that they become dilated, larger-caliber blood vessels that are not under maternal vasomotor control, in order to establish adequate maternal blood flow to the placenta. Initially, the entire gestational sac is covered with villi, but by the end of the first trimester, only the villi at the embryonic pole persist to form the definitive placenta, while the rest regress to form the smooth chorion [1,6].

The villous trophoblast further differentiates into cytotrophoblasts and syncytiotrophoblasts. Cytotrophoblasts are mononuclear cells that serve as progenitor cells, while syncytiotrophoblasts are multinucleated cells that form a continuous layer in direct contact with maternal blood. This structure is crucial for effective nutrient and gas exchange. The syncytiotrophoblast also plays a role in hormone production, synthesizing hormones such as human chorionic gonadotropin (hCG), which is essential for maintaining pregnancy during the early stages [1].

### 2.2. Phases of Implantation

At the time of implantation (7–12 days post-ovulation) the blastocyst consists of the blastocyst cavity, embryoblast (inside) and trophoblast (periphery). The trophoblast is the structure from which the placenta will develop. Implantation is a highly complex, well-organized process in which interactions between the endometrium and embryo are essential. Three stages can be distinguished in the implantation process: apposition, adhesion and invasion [34].

Any dysfunction that occurs during implantation can lead to placental abnormalities, both architectural and morphological. Abnormalities can have long-term consequences, affecting placental function. Impaired placental function is associated with maternal and fetal complications [35].

#### 2.2.1. Apposition and Adhesion

In apposition, initial contact occurs between the blastocyst and the uterine endometrium—a process that determines the site of implantation [36]. Contact becomes more intimate during the adhesion process. In the first two stages of implantation, the blastocyst differentiates and develops, resulting in an internal cell mass (embryo) and trophoblast (placenta). Concurrently, surrounding endometrial stromal cells undergo a transformation process. The transformation of endometrial stromal cells into specialized secretory cells is called decidualization [37,38,39]. The decidua represents the endometrium “specialized” for pregnancy. Directly below the implantation site of the blastocyst, the decidua is called decidua basalis and represents the site where the placenta will develop from the trophoblast.

Optimal blood flow and angiogenesis are necessary conditions for endometrial growth, embryonic growth and placentation. The highest blood flow is found at the uterine fundus, which is the optimal site for implantation [40]. Most embryos (76%) will migrate towards the uterine fundus, while about 12% do not migrate and implantation occurs in other areas of the uterus, and a small proportion of embryos (11%) migrate towards the cervix. It is not yet known whether embryos implant selectively in the endometrium with the highest blood flow or whether embryos implanted in the site with the highest blood flow are the embryos that survive [41].

Placental hypertrophy increases the likelihood that the placenta will be near or above the internal cervical os. Placental hypertrophy can be caused by CO-induced hypoxia in pregnant smokers and can occur in multiple pregnancies, in multiparous women and in older women. In women who have undergone assisted reproduction procedures and in those with endometriosis, abnormal uterine peristalsis may occur due to an increased amplitude and frequency of uterine contractions during the implantation period, which may lead to abnormal implantation near the internal cervical os and consequently a low-lying placenta [42]. The cesarean-section scar may alter myometrial contractility and disrupt contractions during implantation. In this case, implantation in the lower uterine region is more likely, with the development of placenta previa. It can also occur on or in the immediate area of the uterine scar, due to the presence of proteins that enhance endometrial receptivity during normal implantation, such as integrin β3 and leukemia inhibitory factor. These proteins are thought to be overexpressed in the scar area compared to the rest of the uterine cavity [43]. Because there is no blood flow in the scar, this area tends to be hypoxic. However, hypoxia stimulates trophoblast cell proliferation, so the embryo can develop here [44].

Researchers have compared physiological hypoxia in the trophoblast and placenta with pathophysiological hypoxia in tumors. In both cases, the expression of HIF-1α and 2α was identified. This factor regulates cell proliferation, reduces cell death and stimulates vascular remodeling, invasion into local tissues and immune tolerance. Moreover, implantation requires a collagen-rich environment, and with the uterine scar being collagen-rich tissue, the trophoblast can adhere to it [45].

It has been found that the implantation site is to some extent controlled by the concentration of mediators in the endometrium. Different chemokine receptors have been identified on blastocysts and trophoblasts. Cytokines and chemokines are biochemical mediators involved in leukocyte migration and function, as well as in blastocyst migration to and through the endometrium during implantation [46].

#### 2.2.2. Invasion

During invasion, blastocyst trophoblasts differentiate into villous and extravillous trophoblasts. The latter are involved in invasion and either become endovascular trophoblasts, which invade maternal blood vessels, or interstitial trophoblasts, which migrate through the decidua and myometrium to achieve vascular remodeling [47]. Trophoblast invasion becomes pathological in the case of the direct attachment of chorionic villi to the myometrium. Under hypoxic conditions, cytotrophoblast cells normally invade the endometrium, reach the spiral arterioles or maternal arteries and differentiate into a particular vascular phenotype.

Trophoblasts that implant in an avascular scar may invade the uterine wall more deeply. The causes of this phenomenon lie in the absence of underlying tissue with normal vascularization and in the existence of low oxygen tension, which induce the abnormal persistence of the invasive trophoblastic phenotype, resulting in prolonged invasion [44]. In addition, at the level of the scar, the defect in the interface between the endometrium and myometrium leads to the failure of normal decidualization [48].

When the decidua and thus the Nitabuch layer are absent due to the existence of a uterine scar, the villi attach to smooth muscle fibers rather than decidual cells. As a consequence of decreased decidualization, the secretion of anti-invasive factors normally secreted by the decidua will be deficient. Trophoblastic invasion is a proteolysis-controlled process in which metalloprotease-2 (MMP-2) and MMP-9 play a major role [49,50].

The pro or pre–pro inactive forms of MMP-2 and MMP-9 are activated by pro-invasive factors that are initially produced, among others, by NK cells and, in later stages, by decidual cells. If there is a deficiency of MMP-2 or MMP-9, trophoblast invasion is compromised [50]. Physiologically, once trophoblastic invasion is complete, decidual cells inhibit MMP-2 and MMP-9 activity by releasing anti-invasive factors, such as protease inhibitors [49].

Decidual NK (dNK) cells play an extremely important role in regulating trophoblast invasion by controlling EVT function [51]. Controlled placental invasion is the result of balanced interactions between dNK cells and EVTs [52]. dNK cells are weakly cytotoxic cells but are the main producers of cytokines, growth factors and angiogenic factors. They facilitate immune tolerance, implantation, trophoblastic invasion and vascular remodeling. dNK cells are also known as uterine NK (uNK) cells or endometrial NK (eNK) cells [53].

The arterial blood supply to the uterus is provided by the uterine arteries and ovarian arteries. The former will form arcuate arteries from which radial arteries branch off and enter the myometrium. The radial arteries then branch into spiral arteries. This type of blood vessel supplies the intervillous space, thus bathing the chorionic villi in maternal blood. In the uterine arteries, the blood pressure is 80–100 mmHg, while it is 70 mmHg in the spiral arteries and only 10 mmHg in the intervillous space. The two umbilical arteries that arise from the fetal internal iliac arteries carry deoxygenated fetal blood through the umbilical cord to the placenta. The umbilical arteries give rise to chorionic arteries with capillaries inside the villi as their terminus. Nutrients from maternal blood cross the intervillous space, passing through the syncytiotrophoblast, fetal connective tissue and fetal capillary endothelium to reach the fetal blood. Fetal capillaries drain into chorionic veins, which empty into the umbilical vein [54].

### 2.3. General Histologic Organization of the Placenta

The placenta consists of a fetal portion, represented by the chorion (chorionic plate and chorionic villi), and a maternal portion, represented by the basal decidua. The utero-placental circulatory system begins to develop from the ninth day post-conception [1], with the formation of vascular spaces between the decidua and trophoblast known as trophoblastic lacunae. Maternal sinusoids differentiate from capillaries and open into these lacunae. The differential pressure between arterial and venous vessels establishes the direction of blood flow, resulting in primitive utero-placental circulation [6].

The chorionic plate is represented by a thick layer of connective tissue and consists of the amnion, main chorionic villi, chorionic arteries and veins, which are branches of the umbilical arteries and umbilical vein, respectively. Chorionic arteries and veins branch into the arterioles and venules of the main stem villi. These structures protrude into the intervillous space and connect to the basal plate through anchoring villi [55].

The basal plate contains the decidua basalis and is made up of trophoblast cells and decidual cells. In the third trimester of pregnancy, the Nitabuch layer appears, which represents the specific area from which the placenta detaches from the uterus at birth. Placental septa originate from the basal plate, protruding into the intervillous space, creating a canalicular system that delimits 10–40 elevated areas known as cotyledons [56,57]. Endometrial arterioles and venules traverse the basal plate. Exchanges between the maternal and fetal circulatory systems take place between the main stem villi and maternal endometrial arteries and venules, at the level of the intervillous space [34].

After the formation of the blastocyst, the inner cell mass forms the embryoblast, while the peripheral cells form the trophoblast. After day 11, the conceptus continues to grow into the endometrium. Trophoblastic cells proliferate, organize into cords, and form the chorionic villi, which are the functional units of the placenta, i.e., structures in which fetal blood is separated from maternal blood by 3–4 cell layers that make up the placental membrane [1].

**Figure 1:**
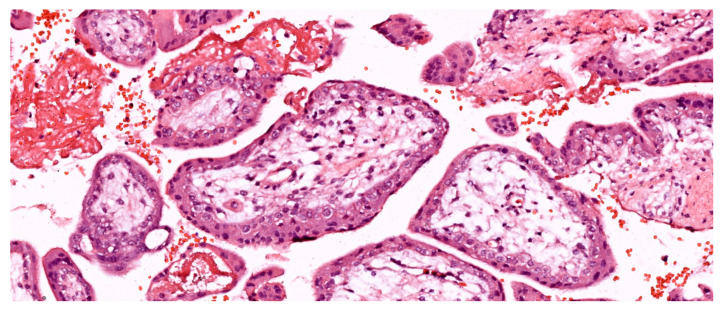
First-trimester chorionic villi (200×), in hematoxylin-eosin (HE) staining.

In the first trimester, villi are composed of peripheral syncytiotrophoblast and inner cytotrophoblast layers (see Figure 1). The growth of the villi primarily depends on the proliferative activity of cytotrophoblastic cells. The core of the villi contains mesenchymal tissue, in which blood vessels develop. In addition, trophoblastic columns form in the deeper implantation zone, extending into the maternal decidua along with the syncytiotrophoblast, and are composed of intermediate trophoblasts (also known as “X cells”). Chorionic villi can be classified into three types based on their morphology:

1. Primary Villi: The smallest type, without extensive branching, composed of a central cytotrophoblast core surrounded by peripheral syncytiotrophoblast. Primary villi start to form between day 11 and day 13 post-conception.
2. Secondary Villi: Branching structures with loose connective tissue in the central axis, forming later in the first trimester.
3. Tertiary Villi: Begin to form at the end of the third week of gestation; they are extensively branched and elongated and contain well-developed blood vessels within the central axis.

**Figure 2:**
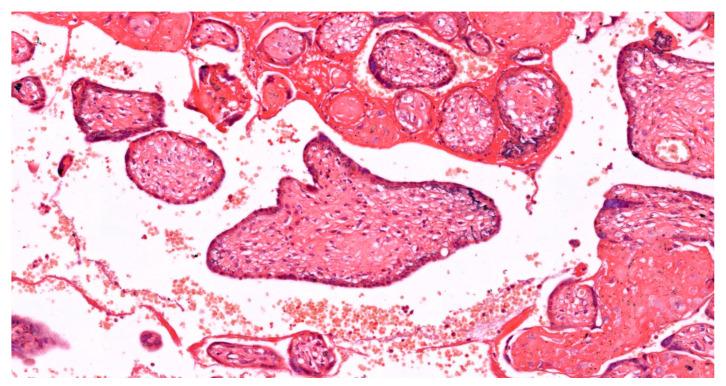
Third-trimester mature chorionic villi (200×), in hematoxylin-eosin (HE) staining.

The loose connective tissue in the villous core includes fibroblasts, Hofbauer cells (macrophages), and blood vessels (see Figure 1). Cytotrophoblasts are composed of individual cuboidal cells. The cytotrophoblastic nuclei are round and euchromatic, and their cytoplasm is pale and contains few organelles. Syncytiotrophoblasts are multinucleated and located at the periphery of the trophoblast. The syncytiotrophoblast cells possess numerous microvilli and pinocytotic vesicles; small, hyperchromatic nuclei; and a cytoplasm rich in well-developed rough endoplasmic reticulum, multiple Golgi complexes, mitochondria, and lipid inclusions. Cytotrophoblasts largely disappear in the second half of pregnancy, whereas syncytiotrophoblasts persist until delivery (see Figure 2).

#### 2.3.1. Structure and Development of Chorionic villi

Considering the developmental stage, villous structure, vascular branches, histological characteristics and cellular components of vascular structures, five types of villi have been described:

1. **Mesenchymal villi**: These represent the most primitive type of villi, from the early stages of pregnancy. The stroma is loose, the capillaries are discrete, there are two layers of surrounding trophoblast cells and a layer of cytotrophoblast cells surrounding the center of the villus and syncytiotrophoblast, arranged on the outer villous surface. Fetal capillaries are poorly developed and never show sinusoidal dilatations. The non-vascularized extremities of mesenchymal villi are called villous buds. The function of mesenchymal villi is very primordial at the beginning of pregnancy. These are the site of villous proliferation and fulfill almost all endocrine activities. As pregnancy progresses, their main function is to support villous growth. In the mature placenta, mesenchymal villi represent less than 1% of the total villous volume [58].
2. **Stem villi**: This type of villi attaches to the chorionic plate and is characterized by a dense fibrous stroma containing both large and small vessels. Vascular structures with smooth muscle develop in the stem chorionic villi. The trophoblast cell layer of stem chorionic villi is partially replaced as pregnancy progresses. The function of stem chorionic villi is to support the structure of the villous “tree”. Endocrine activity and maternal–fetal exchange at the level of stem villi are usually negligible [58].
3. **Immature intermediate villi**: These are peripheral, immature, bulb-shaped continuations of stem villi. They have a looser or reticular stroma. Hofbauer cells, more prominent blood vessels and a discontinuous layer of cytotrophoblast cells are noted in these villi. The outer layer, the syncytiotrophoblast, remains continuous throughout development. Immature intermediate villi form the basis for growth of the villous “tree”. It is considered that maternal–fetal exchange occurs mainly in these villi during the first and second trimesters, until terminal villi differentiate [58].
4. **Mature intermediate villi**: These are long, thin, peripheral branches. This type of villi does not have fetal vessels in the stroma. Terminal villi will arise from mature intermediate villi. The increased number of fetal vessels, providing a large exchange surface, makes them important for feto-maternal exchange [6].
5. **Terminal villi**: These are connected to stem villi. Terminal villi have a grape-like appearance, characterized by a high degree of capillarization and the presence of highly dilated sinusoids. In the term placenta, terminal villi are smaller with less stroma and have a discontinuous cytotrophoblast cell layer and 4–6 fetal capillaries in their cross-section. In terminal villi, fetal capillary vessels and syncytiotrophoblast are separated only by a thin basement membrane, making these villi the most suitable site for maternal–fetal exchange. In the mature placenta, terminal villi represent 40% of the total villous volume of the placenta. Due to their small diameters, the sum of their surfaces represents 50% of the total villous surface area [58]. Terminal villi are considered the functional unit of the placenta. The transfer of electrolytes, O_2_, CO_2_ and nutrients between the mother and fetus occurs at this level [6].

Villous development begins with mesenchymal villi. Until 5 weeks after conception, all chorionic villi are of the mesenchymal type. Subsequently, mesenchymal cells invade these villi, resulting in secondary villi—immature intermediate villi—and placental blood vessels. Placental mesenchymal villi form continuously during pregnancy but predominate in the first and second trimesters. Villous buds transform into immature/mature intermediate villi, and then into terminal villi [59]. Trophoblast budding, proliferation and the formation of trophoblastic protrusions lead to mesenchymal invasion and local fetal angiogenesis. Formation of the fetal vessel villous core and feto-placental blood flow begins approximately at 6–8 weeks after conception [6,59].

At 10 weeks of age, the placenta reaches a weight of about 20 g, and at 20 weeks, the placental weight will be about 150–170 g. A term placenta weighs approximately 500–600 g and has between 15 and 28 cotyledons. The dominant structural unit of the cotyledon is the stem villus. Each cotyledon begins with a stem villus that branches into 3–5 immature/mature intermediate villi, which in turn will give rise to 10–12 terminal villi. Some terminal villi are detached in the intervillous space, while others are connected to the decidua. This attachment provides structural stability to the placenta [6].

#### 2.3.2. Placental Barrier and Materno-Fetal Exchange

The maternal and fetal circulations within the placenta are separated by a specialized structure known as the materno-fetal barrier, which is critical in preventing the direct mixing of maternal and fetal blood while allowing for selective exchange. This barrier consists of several layers:

- Syncytiotrophoblast Layer: This outermost layer is in direct contact with maternal blood. It plays a role in hormone synthesis and transport.
- Cytotrophoblast Layer: A layer of individual cuboidal cells that provide structural integrity and secrete enzymes that aid in remodeling the maternal vasculature.
- Trophoblast Basement Membrane: The extracellular matrix providing support to trophoblastic cells.
- Villous Core Mesenchyme: Contains fibroblasts, Hofbauer cells, and fetal capillaries that transport nutrients and oxygen.
- Endothelial Basement Membrane: A thin extracellular matrix layer that provides a barrier between fetal blood and the surrounding villous core.
- Fetal Capillary Endothelium: The inner layer that lines fetal blood vessels, allowing for nutrient uptake into fetal circulation.

This barrier, although selectively permeable, is less restrictive compared to other biological barriers such as the blood–brain barrier. Substances like nicotine, alcohol, certain heavy metals, and medications (e.g., aminoglycosides) can cross the placental barrier, affecting fetal development. Additionally, some viruses, such as the rubella virus, can pass through, potentially leading to congenital infections [60].

### 2.4. Circulatory Changes and Placental Growth Throughout Pregnancy

Between 10 and 12 weeks of gestation, the trophoblast plugs begin to dissipate, gradually allowing maternal blood to enter the intervillous space [61]. This process results in an increase in placental oxygen tension, which, in turn, stimulates several developmental changes, including fetal vessel branching, cytotrophoblast differentiation into syncytiotrophoblast, and the shift from histotrophic to hemotrophic nutrition [9,61]. Hemotrophic nutrition involves the direct transfer of nutrients from maternal blood to the fetal circulation, facilitated by the extensive network of villous capillaries [61].

During the first trimester, placental development occurs in a relatively hypoxic environment, with maternal blood flow into the intervillous space being restricted by trophoblast “plugs” within the spiral arteries. This low-oxygen environment plays an essential role in promoting trophoblast proliferation and invasion while also protecting the embryo from OS [9]. Histotrophic nutrition, facilitated by uterine gland secretions, is the primary method of nutrient transfer to the embryo during this period. The uterine glands secrete a rich mixture of glycogen, lipids, and other nutrients, which are absorbed by the trophoblasts to support early fetal growth [61].

The hypoxic environment also influences the expression of specific genes in the trophoblast, leading to the production of factors that promote cell proliferation, differentiation, and angiogenesis. HIF is a key transcription factor that is stabilized under low-oxygen conditions and regulates the expression of numerous genes involved in placental development. HIF promotes the formation of new blood vessels (angiogenesis) and enhances trophoblast invasion, which is necessary for establishing an adequate blood supply to the growing placenta [62].

The increase in oxygen saturation during this period is critical for driving the maturation of the placenta [9,61]. It triggers the production of antioxidant enzymes, such as superoxide dismutase (SOD), catalase (CAT), and glutathione peroxidase (GPx), which help to mitigate OS within the developing placenta [63]. This transition also supports the differentiation of cytotrophoblasts into syncytiotrophoblasts, which enhances the placenta’s capacity for nutrient and gas exchange [61]. Additionally, the establishment of maternal–fetal circulation is accompanied by the increased production of angiogenic factors such as VEGF and placenta growth factor (PlGF), which promote the development of the placental vasculature [64].

In the initial stages of development, the blood vessels of the villi anastomose with the embryonic vessels, allowing blood to begin circulating through the primitive cardiovascular system by day 21 post-conception. The spaces between the villi (lacunae) represent the site of metabolic exchange between maternal and fetal circulation [61]. During the first eight weeks, the villi cover the entire surface of the chorion, but only those that come into contact with the basal decidua persist and grow rapidly [56,61]. This portion, which is part of the fetal structures, is called the villous chorion, and the layer from which the villi differentiate is known as the chorionic plate [56]. The basal decidua forms a compact layer, the basal plate, through which spiral arterioles supply blood to the trophoblastic lacunae [11]. The umbilical cord ensures vascular connection between the fetal placenta and the embryo, containing two umbilical arteries and one umbilical vein. The umbilical arteries transport deoxygenated blood to the placenta, while the capillary network within the villi facilitates nutrient and oxygen exchange before the oxygen-rich blood is carried back to the fetus via the umbilical vein [61].

Herein, in the early stages of pregnancy, the circulatory system within the placenta is relatively primitive, and nutrient and oxygen supply to the fetus depend on histotrophic mechanisms [9,61]. However, as the pregnancy progresses, the placental vasculature undergoes significant changes to accommodate the increased demands of the growing fetus [61]. Spiral artery remodeling, carried out by EVTs, ensures that the maternal blood supply to the intervillous space is adequate and that maternal blood flows into the lacunae under reduced pressure, promoting efficient exchange [11].

By the end of the first trimester, the trophoblastic plugs dissolve, and maternal blood flow into the intervillous space becomes more established [9,61]. The development of a complex vascular network in the tertiary villi and the presence of vasculosyncytial membranes in terminal villi facilitate rapid nutrient and gas exchange [56,61]. The placenta continues to grow in size and complexity until around the 36th week of pregnancy, reaching a weight of approximately 500–600 g [56].

The decidua, derived from the endometrial stroma, also plays an important role in supporting placental development. The decidua basalis interacts with the trophoblast to form the maternal component of the placenta, while the decidua capsularis covers the external surface of the growing gestational sac. As the pregnancy advances, the decidua capsularis fuses with the decidua parietalis, obliterating the uterine cavity [65].

The growth of the placenta involves both the hyperplasia and hypertrophy of placental cells [56]. Syncytiotrophoblasts continuously expand through the fusion of underlying cytotrophoblasts, whereas cytotrophoblasts decrease in number as gestation progresses [66]. Mesenchymal villi, which represent the earliest form of villi, differentiate into more complex structures, such as terminal villi, which are highly specialized for nutrient transfer [56].

### 2.5. Functions of the Placenta

The placenta serves several critical functions during pregnancy, primarily acting as the interface for exchange between maternal and fetal circulations. One of its key roles is gas exchange, with the placenta entirely responsible for the transfer of oxygen and carbon dioxide (CO_2_) between mother and fetus, as fetal lungs are non-functional during gestation [67]. Oxygen transfer occurs via passive diffusion, driven by a partial pressure gradient of ~4 kPa between maternal blood in the intervillous space and fetal blood in the umbilical arteries. This process is enhanced by the Bohr effect and the unique properties of fetal hemoglobin. The Bohr effect facilitates oxygen release to the fetus as maternal blood becomes more acidic due to CO_2_ transfer, shifting the maternal oxyhemoglobin dissociation curve to the right. Conversely, as fetal blood releases CO_2_ and becomes more alkaline, its dissociation curve shifts left, further promoting oxygen uptake. This reciprocal process is known as the “double Bohr effect”. Fetal hemoglobin’s higher oxygen affinity, reflected in its left-shifted dissociation curve, also enhances oxygen transfer [67].

CO_2_ transfer from fetus to mother occurs primarily through passive diffusion, based on a partial pressure gradient of ~1.8 kPa. This process is aided by the Haldane effect, whereby deoxygenated blood has an increased capacity to transport CO_2_. As maternal blood releases oxygen and forms deoxyhemoglobin, its ability to carry CO_2_ increases. Simultaneously, fetal blood forms oxyhemoglobin and releases CO_2_ into maternal blood—a phenomenon referred to as the “double Haldane effect” [68].

The placenta also facilitates crucial metabolic transfers. Glucose, the primary energy source for the fetus, is transferred via facilitated diffusion, supplementing the insufficient passive diffusion to meet fetal needs. This is necessary because the fetus has limited gluconeogenesis capacity. Amino acid transfer occurs through active transport mechanisms, utilizing various transporter proteins for anionic, cationic, and neutral amino acids. Most of these are co-transport proteins that exchange sodium for amino acids. Fatty acids and glycerin, crucial for the synthesis of signaling molecules and fetal structures, are primarily transferred by simple diffusion, with some assistance from fatty acid binding proteins. Lipoprotein lipase on the maternal surface of the placenta cleaves lipoproteins into free fatty acids to facilitate this process [69,70].

The transfer of electrolytes, vitamins, and water involves multiple mechanisms. Sodium and chloride ions primarily cross via passive diffusion, although some active transport may occur. Calcium ions, iron, and vitamins are actively transported using carrier molecules. Water transfer occurs through simple diffusion based on hydrostatic and osmotic pressure gradients, potentially aided by water channel proteins in the trophoblast [1].

The placenta also functions as an endocrine organ, producing several important hormones. Human chorionic gonadotropin (hCG) is synthesized by the syncytiotrophoblast in early pregnancy, peaking at about 8 weeks of gestation. It stimulates the corpus luteum to secrete progesterone, crucial for maintaining pregnancy. Human placental lactogen (HPL), also known as human chorionic somatomammotropin, reduces maternal insulin sensitivity, increases maternal glucose levels, stimulates fetal pulmonary surfactant and ACTH production, and promotes mammary gland development for lactation. Placental variant growth hormone (hGH-V) promotes placental growth and stimulates maternal gluconeogenesis and lipolysis, optimizing nutrient availability for the fetus. The placenta also takes over the production of estrogen and progesterone from the corpus luteum after 9 weeks of gestation. Progesterone inhibits uterine contractions and labor onset, while estrogen promotes uterine and mammary gland development [1].

Immunologically, the placenta facilitates the transfer of maternal IgG antibodies to the fetus via pinocytosis. The syncytiotrophoblast has receptors for IgG Fc fragments, allowing for the binding, endocytosis, and release of these antibodies into fetal blood. This transfer begins early in gestation and increases exponentially in the third trimester, providing passive immunity to protect the newborn in the first months of life. However, this process can also allow maternal autoimmune disease antibodies to potentially affect the fetus [54,71].

The placenta also plays a role in drug transfer, which can be both beneficial and harmful [67]. Drug transfer is categorized into three types: complete transfer (e.g., thiopental), where drugs rapidly cross the placenta and achieve balanced maternal–fetal concentrations; excess transfer (e.g., ketamine), resulting in higher fetal blood concentrations; and incomplete transfer (e.g., succinylcholine), where placental crossing is incomplete, leading to higher maternal blood concentrations. The mechanisms of drug transfer include simple diffusion, facilitated diffusion, active transport, and pinocytosis. Factors affecting drug transfer include physical aspects such as placental surface area, blood pH, and uteroplacental blood flow, as well as pharmacological factors like the drug’s molecular weight and protein binding. It is important to note that the period of highest risk for adverse drug effects is during organogenesis in the first trimester, and these effects can be direct or mediated, for example, by altering utero-placental blood flow [72].

Understanding these mechanisms and factors is crucial for assessing the potential risks and benefits of medication use during pregnancy and for developing strategies to optimize maternal treatment while minimizing fetal exposure to potentially harmful substances.

## 3. Definitions and Mechanisms of Oxidative Stress

OS is characterized by the production of an imbalance between the synthesis of free radicals in cells and the body’s ability to detoxify and eliminate these products. ROS are normally generated as byproducts of oxygen metabolism. They have several physiological roles, such as, for example, the role of signaling molecules. But under certain conditions, favored by environmental stress factors (e.g., UV radiation, ionizing radiation, various pollutants, heavy metals, etc.) and xenobiotics (e.g., antiblastic drugs), there is a considerable increase in the production of ROS, resulting in the imbalance that leads to cell and tissue damage: OS. Although considered a pathological condition harmful to the human body, in some cases, OS is used as a therapy for treating certain conditions, such as cancer, with a certain degree of clinical success [73].

### 3.1. Metabolism of Reactive Oxygen Species

Free radicals are molecules that contain oxygen, with an odd number of electrons. The odd number of electrons allows them to react easily with other molecules. Free radicals can cause chain chemical reactions because they react so easily with other molecules. These reactions are oxidation reactions. They can be beneficial or, on the contrary, harmful. Mainly, superoxide radicals, hydrogen peroxide (H_2_O_2_), hydroxyl radicals and singlet oxygen (^1^O_2_) comprise ROS, resulting from metabolic processes [74,75]. Certain reactions or processes such as protein phosphorylation, the activation of certain transcription factors, apoptosis, immune responses and cell differentiation are dependent on adequate ROS production and the maintenance of low intracellular concentrations of ROS [76].

ROS are produced mainly in mitochondria, both under physiological and pathological conditions [59,77], mainly through enzymatic and non-enzymatic reactions. Enzymatic reactions capable of generating ROS are those involved in the respiratory chain, in prostaglandin synthesis, in phagocytosis and in the cytochrome P450 system [78]. Under physiological conditions, the most common free oxygen radical is the superoxide anion (O_2_^−^), with mitochondria being considered the main source. Conversely, the superoxide radical can be formed as a result of cellular respiration, as a result of the action of lipoxygenase (LOX) and cyclooxygenase (COX) during arachidonic acid metabolism, and/or as a result of metabolic processes that occur in endothelial and inflammatory cells [77].

Electron transfer along respiratory chain enzymes is not totally efficient, and the leakage of electrons to molecular oxygen, especially from mitochondrial respiratory chain super-complexes I and III, results in the formation of O_2_^−^. The rate of formation is determined by the number of electrons present on the chain and is therefore increased under conditions of hyperoxia and increased glucose, as in diabetes. Paradoxically, superoxide is also increased under hypoxic conditions, when the availability of oxygen, which acts as the final electron acceptor for mitochondrial respiratory chain super-complex IV, is reduced and causes electron accumulation. Under normal conditions, 2% of the oxygen consumed is converted to O_2_^−^ in mitochondria. Due to its charge, the mitochondrial membrane is impermeable to O_2_^−^ and thus remains in the mitochondrial matrix [77,78].

Similarly, O_2_^−^ can also be generated by electron leakage from the shorter electron transport chain within the endoplasmic reticulum (ER) [41,79]. Disulfide bond formation during protein folding is an oxidative process and, therefore, ~25% of O_2_^−^ in cells is generated in the ER. The amount can increase in cells with high secretory power and also under conditions of ER stress, when repeated attempts to refold cleaved proteins may occur. Other sources of superoxide under physiological conditions are NADPH oxidases (Nox), which generate substantial amounts throughout pregnancy but especially at the beginning of the gestational period, cytochrome P450 and other oxidoreductases. Therefore, various growth factors, drugs and toxins cause an increase in the amount of ROS [76,79].

Under pathological conditions, xanthine dehydrogenase enzyme becomes an important factor. This enzyme degrades purines, xanthine and hypoxanthine to uric acid and, under normal conditions, uses NAD+ as the electron receptor. Under hypoxic conditions, it is proteolytically cleaved to the oxidase form, which donates electrons to molecular oxygen. This enzyme plays a key role in the reperfusion phase of ischemic injury, when its action is amplified by the accumulation of hypoxanthine as a result of ATP decomposition during the hypoxic period.

Superoxide is transformed by SOD enzymes into H_2_O_2_, which is not a free radical, so it is less reactive than O_2_^−^. However, it is included in the term ROS because it is intimately involved in the generation and detoxification of free radicals. Because it is non-polar, it is able to diffuse through cell membranes and therefore acts widely as a second order messenger in signal transduction pathways. In turn, H_2_O_2_ is decomposed into water by CAT and GPx enzymes. It is important that antioxidant enzymes act together because an imbalance of O_2_^−^ and H_2_O_2_ concentrations can lead to the formation of the hydroxyl ion (OH^−^), which is much more dangerous [76,79].

The OH^−^ is the most reactive radical of all free radical species in vivo. It is generated as a result of the reaction between the O_2_^−^ radical and H_2_O_2_, with Fe^2+^ or Cu^+^ as reaction catalysts (Fenton reaction) [77,78]. The OH^−^ has an estimated lifetime of 9–10 s and reacts with any biological molecule in the immediate vicinity—a reaction that is, however, limited by the diffusion process. Because it is so strongly reactive, no pathway for OH^−^ elimination is known.

The excessive generation of O_2_^−^ can also lead to interactions with nitric oxide (NO) to form peroxynitrite (ONOO^−^). Peroxynitrite is a powerful pro-oxidant. Because it is able to diffuse up to 5 μm, it can affect neighboring cells. NO is a radical that plays several important physiological roles and is synthesized as a result of the oxidation reaction of arginine to citrulline, catalyzed by NO synthase [77,78].

Non-enzymatic reactions can also be responsible for free radical production—for example, in situations where oxygen reacts with organic compounds or when cells are exposed to ionizing radiation. Non-enzymatic free radical production can also occur during mitochondrial respiration [5,79,80].

If ROS production increases, harmful effects on important cellular constituents such as proteins, lipids and nucleic acids will begin to appear [81]. There is significant evidence indicating that OS may be responsible, to varying degrees, for the onset and/or progression of several diseases (cancer, diabetes, metabolic disorders, atherosclerosis, cardiovascular diseases, etc.) [82].

The sources of free radical generation are both endogenous and exogenous. Endogenous sources of ROS generation include immune cell activation, inflammation, ischemia, infection, cancer, excessive physical exercise, mental stress and aging. The production of exogenous free radicals can occur as a result of exposure to environmental pollutants, heavy metals (Cd, Hg, Pb, Fe and As), certain drugs (cyclosporine, gentamicin and bleomycin), and chemical solvents; food preparation using certain foods (smoked meat, used oil, fat); and exposure to cigarette smoke, alcohol and radiation. When these exogenous compounds enter the body, they are degraded or metabolized, and free radicals are generated as metabolic products [83,84].

#### 3.1.1. Physiological Roles of Free Radicals

At low or moderate concentrations, free radicals have a number of beneficial roles for the body. They are necessary for the synthesis of certain cellular structures and also for the immune system, to combat pathogens. Phagocytic cells synthesize and store free radicals, to be able to release them when a microbial invasion occurs to destroy pathogens [81]. The most appropriate example is the essential role of ROS in the immune system of patients with granulomatous disease. They cannot produce superoxide radicals, due to a defect in the Nox system, and are thus predisposed to repeated infections in most cases [79].

Free radicals can be produced by non-phagocytic Nox isoforms; in this case, free radicals play a key regulatory role in intracellular signaling cascades between several cell types such as fibroblasts, endothelial cells, vascular smooth muscle cells, cardiac myocytes and thyroid tissue. Nitric oxide (NO-) is the best-known free radical that acts as a signaling molecule. It is an important intercellular messenger necessary for the proper modulation of blood flow, and it is involved in the non-specific defense of the body. The induction of a mitogenic response is another important role of ROS [80].

#### 3.1.2. Pathological Effects of Free Radicals

OS is a phenomenon characterized by a loss of balance between free radical synthesis and protective mechanisms. For example, excess hydroxyl radicals and peroxynitrite can cause lipid peroxidation, resulting in damage to cell membranes and lipoproteins. Consequently, malondialdehyde and conjugated diene, which are cytotoxic and mutagenic compounds, will be formed. Because lipid peroxidation is a chain reaction, it will interfere with the lipid mechanism. OS can cause proteins to undergo conformational changes that could lead to loss or impairment of their enzymatic activity.

OS can also lead to DNA alteration. One of the best-known effects of ROS on DNA is the formation of 8-oxo-2′-deoxyguanosine (8-OHdG), with mutagenic effects and possible effects related to a loss of epigenetic information. Some specialists have proposed that 8-oxo-2′-deoxyguanosine levels in a tissue be used as biomarkers for OS [85].

Uncontrolled, OS can be responsible for inducing several chronic and degenerative conditions, can accelerate the aging process and can cause acute conditions (e.g., stroke) [73].

### 3.2. Antioxidants and Antioxidant Defense Mechanisms

Antioxidants are molecules that can donate an electron to a free radical without losing their stability. Following such a reaction, the free radical stabilizes and becomes less reactive. Oxidant aggression can be inhibited by a series of enzymatic or non-enzymatic phenomena. Cells initiate an antioxidant defense system based mainly on enzymatic components, such as SOD, CAT and GPx, to protect themselves from cell damage induced by ROS [86]. All enzymatic defense systems have a transition metal core, capable of taking on different valences, as it transfers electrons during the detoxification process.

Superoxide is converted to H_2_O_2_ by two isoforms of SOD: manganese SOD, which is limited to mitochondria, and copper-zinc SOD, which is found in the cytosol.

CAT or GPx (tetrameric selenoprotein) break down H_2_O_2_ into water. The activity of GPx depends on the presence of reduced glutathione (GSH) as a hydrogen donor. Glutathione is synthesized in the cytosol from L-glutamate, L-cysteine and glycine and constitutes the main intracellular redox-thiol system. GSH participates in a large number of detoxification reactions forming glutathione disulfide, which is converted back to GSH by the action of glutathione reductase, at the expense of NADPH. NADPH is generated via the pentose phosphate pathway, with glucose-6-phosphate dehydrogenase being the enzyme that catalyzes the first reaction in this chain. Reduced glucose-6-phosphate dehydrogenase activity can compromise GSH concentrations and lead to embryopathy [87].

Non-enzymatic antioxidant systems are represented by ascorbic acid (vitamin C) and α-tocopherol (vitamin E). The two vitamins work together. Ascorbic acid is necessary for the regeneration of reduced α-tocopherol. Thiol compounds, such as thioredoxin, are capable of acting on H_2_O_2_, but in turn require reconversion to the reduced form, via thioredoxin reductase. The inhibition of the Fenton reaction and, implicitly, the production of hydroxyl ions is achieved by the sequestration of free iron ions by ceruloplasmin and transferrin. Polymorphisms of antioxidant enzymes or the dietary restriction of micronutrients, such as selenium, may play an important role in predisposition to OS and the occurrence of pregnancy complications [87,88,89].

### 3.3. The Pro-Oxidant–Antioxidant Balance Concept

Reactivity allows oxygen to participate in high-energy electron transfers and thus supports the generation of large amounts of adenosine-5-triphosphate (ATP) through oxidative phosphorylation. This is necessary to allow for the evolution of complex multicellular organisms, but it also makes it susceptible to attack any biological molecule, whether proteins, lipids or DNA. Consequently, our body is under constant oxidative attack by ROS. There is a complex antioxidant defense system that generally counterbalances this attack, achieving a balance. However, sometimes this balance can be disrupted, which leads to OS. Due to the multiple and diverse effects that oxygen toxicity can have on a cell, OS is best defined, in general terms, as a change in the pro-oxidant–antioxidant balance in favor of the former. This imbalance leads to a series of potential damages. Its role played in the pathophysiology of many disorders, including pregnancy complications, is recognized.

The concept of pro-oxidant–antioxidant balance is essential for understanding OS for several reasons. First, this concept emphasizes that disruption can be caused by changes that can occur on either side of the balance (e.g., abnormally large generation of ROS or deficiencies in antioxidant defense). Secondly, it highlights the homeostatic concentrations of ROS. Although ROS first came to the attention of biologists as potentially harmful by-products of aerobic metabolism, it is now recognized that they play important roles as secondary messengers of several intracellular signaling pathways [79]. Finally, the concept of balance draws attention to the fact that there will be a gradual response to OS. Therefore, minor disturbances of the balance can lead to homeostatic adaptations in response to changes in the proximal environment, while major disruptions can lead to irreparable damage and cell death. The boundary between physiological and pathological changes is difficult to specify.

There are many potential sources of ROS, and their relative contributions will depend on the prevailing environmental circumstances. Because ROS reactions are often diffusion-limited, effects on cell function largely depend on biomolecules in the immediate vicinity. Different lesions will therefore generate different outcomes.

Another feature of OS that shapes its clinical manifestation is that it rarely occurs in isolation. It is now appreciated that complex interactions occur between OS and other forms of cellular stress, such as ER stress. The close interactions between ROS, mitochondrial function and the ER, mediated through Ca^2+^ release, can constitute a feed-forward system [90]. Recent research has highlighted that the ER represents a major center for coordinating cellular responses to a variety of stressors. This is largely due to the fact that protein synthesis accounts for approximately 30% of a cell’s energy expenditure and therefore synthesis must be finely adapted to the availability of oxygen and nutrients.

Under stress conditions, the unfolded protein response (UPR) (a set of signaling pathways aimed at restoring homeostasis) aims to restore homeostasis through a set of coordinated responses that reduce misfolded proteins. Thus, firstly, the influx of new proteins will be blocked by the phosphorylation and inhibition of the eukaryotic initiation factor eIF2α, which regulates translation initiation. Subsequently, the expression of ER proteins, namely Glucose-Regulated Protein (GRP) 78 and 94, increases to try to sequester or refold unfolded proteins. Thirdly, the synthesis of ER cisternae increases. Finally, the ER-associated degradation mechanism is stimulated. If these phenomena fail and ER stress persists, then UPR will activate the apoptotic cascade through an increased expression of C/EBP homologous protein, to eliminate the cell [91].

Another aspect of ER stress that is of particular importance for pregnancy is the relationship with pro-inflammatory pathways. The activation of pro-inflammatory pathways can occur through at least two mechanisms. The first involves the nuclear factor kappa B (NF-κB) pathway. One of the three signaling transducer pathways activated during UPR initiation, the inositol-1-dependent protein (Ire1) pathway, has dual action. Ire1 contains an endoribonuclease, which, when activated, splices X-box binding protein 1 (XBP-1) pre-mRNA to produce the transcription factor, XBP-1, which stimulates the transcription of genes that control misfolded protein repair and ER biogenesis. Ire1 also contains a Ser/Thr kinase that is capable of activating the NF-κB pathway by phosphorylating the inhibitor of nuclear factor kappa B (IκB), as well as the p38 and stress-activated protein kinases (SAPK)/Jun amino-terminal kinases (JNK) pathways via apoptosis signal-regulating kinase 1 (ASK1) [91]. The second mechanism is not directly related to UPR activation; it involves proteins whose structure is similar to that of one of the other signal transduction proteins, thus activating the transcription factor [92].

## 4. Sources of Oxidative Stress During Pregnancy

OS plays a crucial role in placental development and function throughout pregnancy. While excessive OS can lead to pathology, a certain level of ROS signaling is essential for normal placental development and function. The placenta is exposed to various sources of OS throughout gestation. Understanding these sources is crucial for elucidating the mechanisms of placental development and pathology (see Table 1).

One of the primary sources of OS in the placenta is the dramatic fluctuation in oxygenation levels that occurs during development. As described earlier, the first-trimester placenta develops in a relatively hypoxic environment (<20 mmHg oxygen partial pressure). Around 10–12 weeks, the onset of maternal blood flow causes a 2–3-fold increase in oxygen levels. This transition exposes placental tissue to a relative hyperoxia, which can lead to an increased production of ROS. The syncytiotrophoblast layer is particularly vulnerable, as it is the first tissue exposed to maternal blood and has relatively low levels of antioxidant enzymes early in pregnancy. Later in gestation, the intermittent perfusion of the intervillous space due to uterine contractions or variations in blood flow can cause repeated cycles of hypoxia–reoxygenation. This ischemia-reperfusion-type injury is a potent stimulus for ROS generation [93,94].

The placenta is a highly metabolically active organ with abundant mitochondria, particularly in the syncytiotrophoblast. Mitochondrial electron transport is a major source of superoxide radicals under normal conditions, with an estimated 1–2% of oxygen consumed being converted to O_2_^−^. This baseline ROS production can be exacerbated by various pregnancy complications. For example, in PE, there is evidence of mitochondrial dysfunction and increased mitochondrial lipid peroxidation in the placenta.

NOX enzymes are dedicated ROS-producing enzymes that play important roles in cell signaling and host defense. Multiple NOX isoforms are expressed in the placenta, including NOX1, NOX2, NOX4, and NOX5. NOX enzymes contribute to physiological ROS signaling in the placenta, regulating processes like trophoblast differentiation and angiogenesis. However, excessive NOX activation, as seen in some pregnancy complications, can lead to oxidative damage [95,96,97,98].

Xanthine oxidase is another important source of ROS, particularly during ischemia-reperfusion events. Under hypoxic conditions, xanthine dehydrogenase is converted to xanthine oxidase. Upon reperfusion and reoxygenation, xanthine oxidase uses oxygen as an electron acceptor, generating superoxide radicals [99,100].

Nitric oxide synthase (NOS) enzymes normally produce NO, an important signaling molecule in vascular function. However, under certain conditions such as tetrahydrobiopterin deficiency, NOS can become “uncoupled” and produce O_2_^−^ instead of NO. This not only increases ROS production but also reduces the bioavailability of NO [101,102].

The placenta is exposed to high concentrations of hemoglobin (Hb) from maternal blood in the intervillous space. The auto-oxidation of Hb can generate superoxide radicals. Furthermore, free Hb released from damaged erythrocytes can act as a pro-oxidant [103,104].

Maternal immune cells, particularly macrophages and neutrophils, are present in the placental bed and can be a significant source of ROS. Activation of these cells, as occurs in infection or in pregnancy complications like PE, can lead to a respiratory burst and the release of large amounts of ROS [105,106].

Finally, various external factors (maternal smoking, pollution, radiation, medications, alcohol) [107,108] and pre-existing maternal conditions (diabetes mellitus, obesity hypertensive disorders, advanced age) [109,110] can increase placental OS. Understanding these diverse sources of OS is crucial for interpreting placental pathology and developing targeted interventions to mitigate oxidative damage. OS can induce a series of cellular responses, depending on the severity of the injury and the compartment in which ROS are generated. The close interaction between OS and ER stress is important when analyzing therapeutic options, as the benefits will be low if the approach targets only one type of stress.

## 5. Oxidative Stress and Placental Development

OS manifests at the maternal–fetal interface from the beginning of pregnancy. It plays a physiological role in placental development, as well as in the pathophysiology of complications: spontaneous abortion, PE, IUGR and the premature rupture of membranes [10]. However, ROS also play crucial physiological roles in normal placental development and function, i.e., they serve important signaling functions, promoting trophoblast proliferation, differentiation, and angiogenesis. A certain level of OS appears to be necessary for proper placentation. The low oxygen tension in early pregnancy helps to limit ROS production, reducing the risk of oxidative damage to developing tissues. However, excessive OS, if not adequately controlled, can contribute to placental pathologies.

In early placental development, low levels of ROS act as important signaling molecules. They contribute to trophoblast differentiation, promoting the conversion of cytotrophoblasts into syncytiotrophoblast through increased mitochondrial activity and ROS production. Moderate ROS levels enhance the invasive capacity of EVT cells, partly mediated through the activation of MMPs. ROS signaling is also involved in the expression of angiogenic factors like VEGF, promoting placental vascular development. Additionally, ROS play a role in oxygen sensing mechanisms, allowing the placenta to adapt to changes in oxygen partial pressure [31].

The human placenta is unique, in that chorionic villi initially form on the entire surface of the chorionic sac. However, starting from the end of the first trimester of gestation, the villi on the superficial pole regress, thus outlining the definitive discoid placenta. It is now considered that OS plays a central role in this process. Because this regression occurs in all pregnancies, it can be considered physiological.

It is accepted that placental development takes place at a relatively low oxygen concentration, being supported more by endometrial gland secretions than by maternal circulation [111]. Researchers suggest that this environment protects the developing embryo from teratogenesis mediated by oxygen free radicals [112]. Blood is prevented from entering the intervillous space of the placenta by groups of EVT cells that block (like plugs) the spiral uterine arteries, as part of the physiological conversion process in the first trimester of pregnancy. Intraplacental maternal circulation is fully established only towards the end of the first trimester of pregnancy, when these “plugs” are dislodged by a currently unknown mechanism. Ultrasonographic evidence has shown that circulation begins preferentially at the periphery of the placenta, where trophoblast invasion is least extensive and subsequently progressively extends towards the central region [113,114].

The onset of circulation is associated with a tripling of the oxygen concentration at the placental level. This will stimulate higher rates of ROS generation, especially in the critical syncytiotrophoblast layer, where concentrations of antioxidant enzymes, copper-zinc SOD and CAT are low. Consequently, villi taken from the peripheral region of the placenta have high levels of the HSP70 chaperone, nitrotyrosine residues, which indicate peroxynitrite formation and reveal degenerative morphological changes in syncytiotrophoblast, compared to specimens taken from the central region. Molecular evidence confirms that this apoptotic cascade is activated in peripheral villi and that this would be sufficient to explain their regression [115,116,117].

As pregnancy progresses, there is a gradual increase in placental OS markers. This is considered a normal physiological process related to increased placental metabolism and preparation for parturition. OS contributes to increased apoptosis in the syncytiotrophoblast, promoting the normal turnover of this layer. It also activates inflammatory pathways involved in the initiation of labor and contributes to the maturation of fetal organs, particularly the lungs, in preparation for extrauterine life [63].

To balance the physiological roles of ROS with their potential for damage, the placenta develops robust antioxidant defenses. These include enzymatic antioxidants such as SOD, CAT, and GPx, as well as non-enzymatic antioxidants like vitamin C, vitamin E, and GSH. The expression and activity of these antioxidant systems increase as gestation progresses, paralleling the increase in OS [4].

Redox signaling continues to play important roles in placental function throughout pregnancy. ROS modulate the activity of various vasoactive factors, regulating placental blood flow. Certain nutrient transport systems are redox-sensitive. ROS influence the synthesis and release of placental hormones and are involved in the placenta’s immunomodulatory functions [109].

## 6. Oxidative Stress and Placental Pathology

When the balance between pro-oxidant factors and antioxidant defenses is disrupted, OS can lead to various forms of placental pathology. Excessive OS in early pregnancy can inhibit cytotrophoblast fusion and the differentiation into syncytiotrophoblasts, leading to a reduction in the syncytiotrophoblast layer crucial for nutrient transport and hormone production. While moderate ROS promote invasion, excessive OS impairs the invasive capacity of EVTs, resulting in shallow placentation and the inadequate remodeling of spiral arteries. OS can also disrupt the delicate balance of pro- and anti-angiogenic factors, leading to abnormal placental vascular development [31]. These early disruptions can set the stage for later pregnancy complications such as PE and IUGR.

Chronic oxidative stress can lead to various structural alterations in the placenta. These include villous changes such as increased syncytial knots, the thinning of the syncytiotrophoblast layer, fibrinoid necrosis, and villous fibrosis. Vascular changes may include the reduced vascularization of terminal villi, fibrin deposition in vessel walls, and thrombosis. Increased intervillous fibrin deposition and placental infarctions are also observed. These structural changes reduce the functional capacity of the placenta and can be observed histologically in placentas from complicated pregnancies [118].

Furthermore, OS-induced damage leads to various functional impairments. Oxidative damage to syncytiotrophoblast membranes and transport proteins can impair the transfer of crucial nutrients like glucose and amino acids. OS can disrupt the synthesis and release of placental hormones, including hCG and placental lactogen. It can increase the permeability of the placental barrier, potentially allowing harmful substances to reach the fetus. OS also contributes to endothelial dysfunction and altered vascular reactivity, reducing placental blood flow [105].

### 6.1. Placental Adaptations to Oxidative Stress

The placenta has evolved various adaptive mechanisms to cope with oxidative challenges. One primary response is the upregulation of antioxidant systems. This includes an increased expression and activity of SOD, enhanced CAT activity, and the upregulation of GPx and glutathione reductase. Non-enzymatic antioxidants are also boosted, with the increased synthesis of glutathione and enhanced uptake and utilization of vitamins C and E. The nuclear factor erythroid 2-related factor 2—Kelch-like ECH-associated protein 1 (Nrf2-Keap1) system, a key regulator of cellular antioxidant responses, is activated under OS, with Nrf2 translocating to the nucleus to activate the transcription of numerous antioxidant genes [4].

Metabolic adaptations also occur in response to OS. Under hypoxic conditions, the placenta can shift to anaerobic metabolism, increasing anaerobic glycolysis to reduce reliance on oxidative phosphorylation and minimize mitochondrial ROS production. There is also an increase in pentose phosphate pathway activity, which provides NADPH crucial for maintaining glutathione in its reduced form. Changes in mitochondrial dynamics, including alterations in fission and fusion, can help segregate damaged mitochondria and maintain overall mitochondrial function [96].

**Figure 3:**
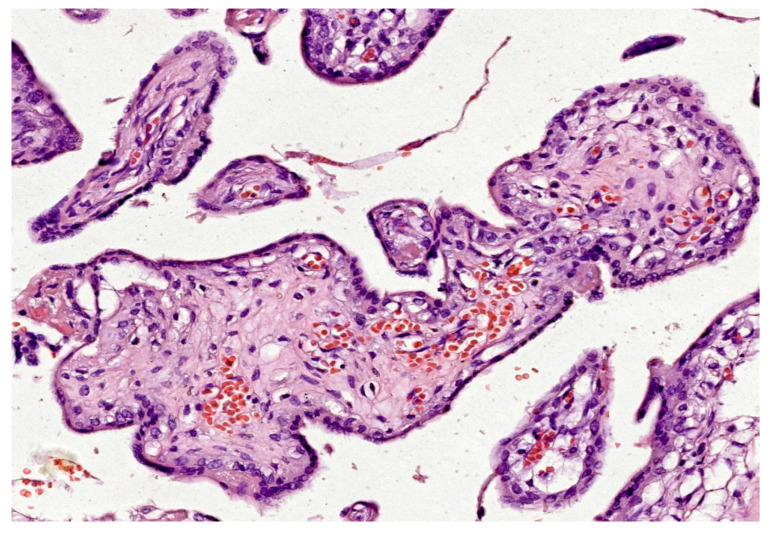
First-trimester chorionic villi (200×), in hematoxylin-eosin (HE) staining, demonstrating enlargement, chorangiosis and accelerated villous maturation.

Structural adaptations are another response to OS. While excessive syncytial knots are pathological, a moderate increase may represent an adaptive response to shed damaged syncytiotrophoblasts. Chorangiosis, or the increased vascularization of terminal villi, can occur in response to hypoxia, potentially improving oxygen delivery (see Figure 3). The thickening of the vasculosyncytial membrane may serve as a protective barrier against oxidative insults [118].

The placenta can also modulate angiogenic factors in response to OS, i.e., stimulate VEGF production, promoting angiogenesis to improve blood flow. Changes in the ratio of angiopoietin-1 to angiopoietin-2 can modify vascular stability and permeability. The induction of PlGF can promote vascular growth and maturation [31].

Various cellular stress response pathways are activated under oxidative conditions. The heat shock response induces heat shock proteins (HSPs), which act as molecular chaperones, helping to maintain protein folding and prevent aggregation. The unfolded protein response (UPR) is activated to manage endoplasmic reticulum stress, which often accompanies OS. DNA repair mechanisms are upregulated to address oxidative DNA damage [109].

Autophagy is upregulated in response to OS and serves several protective functions. It facilitates the removal of damaged organelles, particularly mitochondria (mitophagy), and the recycling of oxidized proteins, and provides nutrients during periods of stress. While excessive inflammation is detrimental, the controlled activation of inflammasomes can have adaptive functions, promoting the removal of damaged cells, stimulating repair and regeneration processes, and enhancing antioxidant responses [96].

The modulation of gasotransmitter production can also be an adaptive response. Increased NO production can improve blood flow and have antioxidant effects. H_2_S signaling can support mitochondrial function and have cytoprotective effects. CO production has anti-inflammatory and vasodilatory properties [63].

The placenta can release extracellular vesicles (EVs) as an adaptive response. These EVs can carry antioxidant enzymes, potentially extending antioxidant capacity. They facilitate communication with maternal tissues to modulate systemic responses and may play a role in fetal programming to enhance offspring resilience [105].

Epigenetic changes can occur as an adaptive response to OS. These include DNA methylation changes to modify gene expression, histone modifications to alter chromatin structure, and changes in microRNA expression to fine-tune protein levels. These epigenetic adaptations may not only help the placenta cope with current stressors but could also potentially prepare the fetus for a challenging postnatal environment [4].

Understanding the balance between physiological and pathological OS, as well as the adaptive responses of the placenta, is crucial for developing interventions that support placental function without disrupting important physiological adaptations. This knowledge may lead to improved strategies for managing pregnancy complications and optimizing long-term health outcomes for both mother and child.

### 6.2. Associations with Specific Placental Pathologies

OS has been implicated in several specific placental pathologies, as summarized in Table 2. In maternal vascular malperfusion (MVM), OS plays a central role, impairing trophoblast invasion, causing endothelial dysfunction in spiral arteries, and increasing the apoptosis of villous trophoblasts. In fetal vascular malperfusion (FVM), OS can exacerbate endothelial damage, leading to thrombosis, and increase inflammation and coagulation. In chronic villitis, OS can both cause and be exacerbated by inflammatory cell infiltration. In chorioamnionitis, infection-induced inflammation leads to increased ROS production through the activation of maternal neutrophils and direct induction by bacterial products. In placental abruption, OS can predispose to abruption and is further increased by the resulting hemorrhage and ischemia reperfusion [96].

### 6.3. Long-Term Materno-Fetal Consequences

The effects of placental OS extend beyond pregnancy, potentially influencing long-term health outcomes for both mother and offspring, as summarized in Table 3. This concept aligns with the Developmental Origins of Health and Disease (DOHaD) hypothesis, which posits that in utero exposures can program lifelong health trajectories.

## 7. Clinical Complications and Oxidative Stress Biomarkers

OS plays a significant role in placental pathology and has been implicated in various pregnancy complications [3,31]. This section reviews key biomarkers of OS that have been studied in relation to placental dysfunction, with promising clinical applications for the early detection of pregnancy complications.

Lipid peroxidation, a consequence of OS, results in the formation of several measurable products, including malondialdehyde, F2-isoprostanes, and 4-hydroxynonenal (4-HNE) [154,155]. These biomarkers are frequently elevated in placentas from complicated pregnancies, particularly in cases of PE and IUGR [13]. Their presence indicates oxidative damage to cellular membranes, which can compromise placental function.

OS can also lead to significant protein modifications, detectable through various markers such as protein carbonyls, advanced oxidation protein products (AOPPs), and nitrotyrosine [156,157,158]. Increased levels of these markers indicate oxidative damage to placental proteins, potentially impairing their function. Such impairment may contribute to placental insufficiency and associated pregnancy complications.

Nucleic acids are likewise susceptible to oxidative damage, which can be assessed through specific biomarkers, including 8-OHdG and 8-hydroxyguanosine. These markers are often elevated in various placental pathologies, indicating oxidative damage to DNA and RNA. Such damage may have implications for placental gene expression and cellular function [159,160,161,162].

The body’s antioxidant defense system plays a crucial role in mitigating OS. Alterations in this system can be indicative of OS and are often observed in placental pathologies [159,161]. These changes may include reduced levels or activity of antioxidant enzymes such as SOD, CAT, and GPx, as well as decreased levels of non-enzymatic antioxidants like vitamin C, vitamin E, and glutathione. These changes in antioxidant status can reflect the placenta’s compromised ability to counteract OS.

OS can modulate the expression and activation of redox-sensitive transcription factors, which play key roles in cellular responses. Nrf2, a key regulator of antioxidant responses, and NF-κB, involved in inflammatory responses, are two such factors [163,164]. An altered expression or activation of these factors can indicate OS-induced changes in gene expression, potentially affecting placental function and adaptation.

Mitochondria are both sources and targets of ROS. Markers of mitochondrial dysfunction, such as an altered mitochondrial DNA copy number and changes in electron transport chain enzyme activities, can provide insights into OS-induced damage [165,166]. These markers can reflect OS-induced mitochondrial damage, which may compromise placental energy metabolism.

Gaseous signaling molecules, or gasotransmitters, play important roles in placental function and can be affected by OS. These include NO metabolites, H_2_S levels, and CO production [27,102]. Alterations in these signaling molecules can indicate OS and vascular dysfunction in the placenta.

In Table 4, we summarize the key OS-associated biomarkers discussed in this section.

The assessment of these diverse biomarkers provides valuable insights into the extent and nature of OS in placental pathology. Their measurement and interpretation can contribute to our understanding of the pathophysiology of pregnancy complications and may inform potential therapeutic strategies. Table 5 provides a comprehensive overview of the role of OS in major pregnancy complications, highlighting key findings and potential biomarkers for each condition.

PE, a hypertensive disorder of pregnancy, is characterized by placental ischemia-reperfusion injury, endothelial dysfunction, and systemic inflammation [31]. The placenta exhibits increased OS markers and reduced antioxidant capacity. A key finding is the elevation of circulating anti-angiogenic factors. Potential biomarkers for PE include the sFlt-1/PlGF ratio, malondialdehyde, 8-isoprostane, and nitrotyrosine [167].

IUGR is associated with impaired placental development, reduced nutrient transport, and mitochondrial dysfunction [118]. The placenta shows increased oxidative damage, altered gene expression, and reduced antioxidant enzymes. Biomarkers for IUGR include F2-isoprostanes, protein carbonyls, 8-OHdG, and indicators of mitochondrial DNA damage [4,168].

In gestational diabetes mellitus (GDM), hyperglycemia-induced ROS production, mitochondrial dysfunction, and advanced glycation end products contribute to OS. Key findings include increased lipid peroxidation, reduced antioxidant defenses, and altered placental insulin signaling. Potential biomarkers for GDM are 8-isoprostane, advanced glycation end products, and the reduced glutathione/oxidized glutathione ratio [169].

Preterm birth is associated with inflammation-induced OS, the premature rupture of membranes, and the activation of labor pathways. Increased OS markers in amniotic fluid, reduced antioxidant capacity in maternal circulation, and oxidative damage to fetal membranes are observed. F2-isoprostanes in amniotic fluid, myeloperoxidase, and matrix metalloproteinases serve as potential biomarkers [170].

Recurrent pregnancy loss involves impaired trophoblast invasion, endothelial dysfunction, and oxidative damage to oocytes/embryos. Increased OS markers in maternal circulation, reduced antioxidant capacity, and oxidative DNA damage in placental tissues are key findings. Biomarkers include 8-OHdG, lipid hydroperoxides, and total antioxidant capacity [171].

Placental abruption is characterized by acute ischemia-reperfusion injury, the activation of inflammatory cascades, and systemic OS in severe cases. Increased markers of oxidative damage in placental tissue, elevated inflammatory mediators, and potential alterations in coagulation factors are observed. Malondialdehyde, protein carbonyls, and inflammatory cytokines (e.g., IL-6, TNF-α) serve as potential biomarkers [172].

## 8. Emerging Therapeutics Targeting Oxidative Stress in Placental Disorders

Given the significant role of OS in various placental disorders, numerous therapeutic approaches have been investigated to mitigate oxidative damage and improve placental function. Table 6 reviews both established and experimental interventions.

## 9. Conclusion and Future Perspectives

This comprehensive review has explored the intricate relationship between OS and placental morphopathology, highlighting its crucial role in both normal placental development and various pregnancy complications. Several key themes have emerged:

1. The placenta exists in a delicate redox balance throughout gestation, with physiological levels of ROS playing important signaling roles in placental development and function [195,196].
2. A disruption of this balance, leading to OS, is implicated in a wide range of placental pathologies and pregnancy complications, including PE, IUGR, gestational diabetes, and preterm birth.
3. OS induces structural and functional changes in the placenta, affecting trophoblast differentiation, vascular development, nutrient transport, and hormone production.
4. The placenta has evolved various adaptive mechanisms to cope with oxidative challenges, but these can be overwhelmed in pathological conditions.
5. The effects of placental OS extend beyond pregnancy, potentially influencing long-term health outcomes for both mother and offspring through epigenetic modifications and other programming mechanisms.
6. While antioxidant therapies have shown promise in preclinical studies, translation to effective clinical interventions has been challenging, highlighting the complexity of redox biology in pregnancy.

Looking to the future, several key areas warrant further investigation:

- Improved biomarkers: Development of more specific and sensitive biomarkers for placental OS could enable the earlier detection of at-risk pregnancies and more targeted interventions [197].
- Personalized approaches: Given the heterogeneity of placental disorders, personalized medicine approaches that consider individual genetic, environmental, and clinical factors may be necessary to effectively manage OS.
- Targeted therapies: Novel drug delivery systems that can specifically target the placenta could improve the efficacy of antioxidant therapies while minimizing systemic effects.
- Timing of interventions: Better understanding the critical windows of susceptibility to OS during placental development could inform the optimal timing of preventive or therapeutic interventions.
- Long-term follow-up: Extended follow-up studies of both mothers and offspring are needed to fully elucidate the long-term consequences of placental OS and evaluate the efficacy of interventions.
- Integration of multi-omics data: Combining genomic, epigenomic, transcriptomic, proteomic, and metabolomic data could provide a more comprehensive understanding of the complex interplay between OS and placental function.
- Advanced imaging techniques: The development of non-invasive imaging methods to assess placental redox status in vivo could revolutionize the monitoring and management of placental health.
- Microbiome interactions: Exploration of the potential role of the placental and maternal microbiome in modulating OS and placental function [198].
- Environmental influences: Further investigation of how environmental factors (e.g., air pollution, endocrine disruptors) influence placental OS and the development of strategies to mitigate these effects [199].
- Therapeutic potential of gasotransmitters: Deeper exploration of the therapeutic potential of gasotransmitters (NO, CO, H_2_S) in managing placental OS and vascular function.

In conclusion, OS plays a central role in placental biology, with significant implications for pregnancy outcomes and long-term health. As our understanding of the complex interplay between oxidative stress and placental function deepens, we are poised to make significant advances in the prevention, diagnosis, and treatment of pregnancy complications. The multifaceted nature of OS in placental pathology necessitates a holistic approach, integrating basic science discoveries with clinical applications.

## Figures and Tables

**Table 1:** Sources of oxidative stress in the placenta during pregnancy.

Source	Description	Key Features
Fluctuations in Oxygenation [93,94]	- Dramatic changes in O_2_ levels during pregnancy - Transition from hypoxic to normoxic/hyperoxic environment	- First trimester: <20 mmHg SpO_2_ - After 12 weeks: >50 mmHg SpO_2_ - Syncytiotrophoblast particularly vulnerable
Mitochondrial Sources [95,96]	- Electron leakage from respiratory chain - Primary source of superoxide radicals	- 1–2% of O_2_ consumed converted to superoxide - Increased in conditions like PE
NADPH Oxidases [97,98]	- Dedicated ROS-producing enzymes - Multiple isoforms expressed in placenta	- NOX1, NOX2, NOX4, NOX5 present - Involved in physiological signaling/pathology
Xanthine Oxidase [99,100]	- Activated during ischemia-reperfusion events - Converts xanthine to uric acid, producing ROS	- Increased activity in PE - Contributes to oxidative damage during blood flow fluctuations
Uncoupled NO Synthase [101,102]	- NOS produces O_2_^−^ instead of NO when uncoupled - Often due to tetrahydrobiopterin deficiency	- Reduces NO bioavailability - Contributes to endothelial dysfunction
Auto-oxidation of Hemoglobin [103,104]	- Hb from maternal blood in intervillous space - Can release free iron, a potent pro-oxidant	- Increased in conditions with placental damage - Contributes to lipid peroxidation
Inflammatory Cells [105,106]	- Maternal immune cells in placental bed - Can produce ROS through respiratory burst	- Increased in inflammatory conditions - Contribute to oxidative damage in infection/PE
Environmental Factors [107,108]	- External sources increasing placental OS	- Maternal smoking - Air pollution - Radiation exposure - Certain medications - Alcohol consumption
Maternal Conditions [109,110]	- Systemic conditions affecting placental oxidative balance	- Diabetes mellitus - Obesity - Advanced maternal age - Hypertensive disorders

NADPH: Nicotinamide Adenine Dinucleotide Phosphate; NOX: NADPH Oxidase; NO: Nitric Oxide; NOS: Nitric Oxide Synthase; O_2_: oxygen; SpO_2_: partial pressure of O_2_; ROS: Reactive oxygen species; O_2_^−^: Superoxide anion; OS: Oxidative stress.

**Table 2:** Association between oxidative stress and specific placental pathologies [119,120,121,122,123,124,125,126,127].

Pathology	Role of Oxidative Stress	Histological Features
Maternal Vascular Malperfusion [119]	- Impairs trophoblast invasion - Causes endothelial dysfunction - Increases villous trophoblast apoptosis	- Decidual arteriopathy - Accelerated villous maturation - Distal villous hypoplasia - Increased syncytial knots - Infarction and retroplacental hemorrhage
Fetal Vascular Malperfusion [120,121]	- Causes endothelial damage leading to thrombosis - Increases inflammation and coagulation	- Villous stromal-vascular karyorrhexis - Avascular villi - Thrombosis in fetal vessels
Chronic Villitis [122,123,124]	- Inflammatory cell infiltration increases ROS production - Promotes further inflammation	- Lymphohistiocytic infiltrate in villi - Destruction of villous architecture - Fibrinoid necrosis
Chorioamnionitis [125]	- Activates maternal neutrophils leading to respiratory burst - Bacterial products directly induce ROS production	- Neutrophil infiltration in chorion and amnion - Funisitis - Potential secondary fetal vascular malperfusion
Placental Abruption [126]	- Oxidative damage predisposes to abruption - Hemorrhage and ischemia-reperfusion increase OS	- Retroplacental hematoma - Compressed villi adjacent to abruption site - Secondary ischemic changes in affected regions
Gestational Trophoblastic Diseases [127]	- Implicated in abnormal trophoblast proliferation and differentiation - Alters angiogenesis	- Hydropic swelling of villi - Trophoblast hyperplasia - Abnormal vasculature

ROS: Reactive oxygen species; OS: Oxidative stress.

**Table 3:** Long-term consequences of oxidative stress (OS) during pregnancy [128,129,130,131,132,133,134,135,136,137,138,139,140,141,142,143,144,145,146,147,148,149,150,151,152,153].

Category		Consequence	Mechanism	Summary of Evidence
Maternal	CV Risk	↑ Risk of CV disease	Persistent endothelial dysfunction, OS and inflammation	2–4 fold ↑ risk of future CV events in women with history of PE [128,129].
		Hypertension	Vascular remodeling, altered renin-angiotensin system	↑ risk of chronic hypertension after hypertensive disorders of pregnancy [130].
	Metabolic Risk	Type 2 diabetes	Persistent β-cell dysfunction, insulin resistance	Up to 7-fold ↑ risk after gestational diabetes [131].
		Metabolic syndrome	Persistent OS, inflammation	↑ prevalence after PE and gestational diabetes [132].
	Renal Risk	Chronic kidney disease	Persistent renal endothelial dysfunction, microvascular damage	4–5 fold ↑ risk after PE [133].
	Cognitive Function	Cognitive decline and dementia	Cerebrovascular effects of OS and inflammation	↑ risk of vascular dementia after PE [134].
Offspring Programming	CV Risk	Hypertension	Altered vascular development, epigenetic changes in CV regulatory genes	↑ blood pressure in childhood and young adulthood after exposure to PE [135].
		↑ CV disease risk	Early vascular dysfunction, altered lipid metabolism	↑ CV risk factors in offspring exposed to maternal obesity or diabetes [136].
	Metabolic Risk	Obesity	Altered hypothalamic circuits regulating appetite and metabolism	↑ risk of childhood obesity after exposure to maternal obesity or gestational diabetes [137].
		Type 2 diabetes	Impaired pancreatic β-cell development and function	↑ risk in offspring exposed to maternal diabetes [138].
	Neurodevelopmental Outcomes	Autism spectrum disorders	Oxidative damage to developing neurons, altered neurotransmitter systems	↑ risk after exposure to PE and other pregnancy complications [139].
		ADHD	Altered neurodevelopment due to OS and inflammation	Association between maternal PE and offspring ADHD [140].
		Cognitive impairment	Oxidative damage to developing brain, altered cerebral blood flow	Lower cognitive scores in children born after preeclamptic pregnancies [141].
	Immune Function	Allergic diseases	Altered immune system development due to OS	↑ risk of asthma and allergies in offspring exposed to maternal obesity and gestational diabetes [142].
		Autoimmune diseases	OS-induced changes in T cell differentiation	Some evidence for ↑ risk of type 1 diabetes after PE [143].
	Renal Function	Reduced nephron population	OS impact on nephrogenesis	Reduced kidney size and function in offspring exposed to PE or IUGR [144].
Epigenetic Mechanisms		DNA methylation changes	OS stress-induced alterations in methylation patterns	Altered methylation in genes related to metabolism and vascular function in cord blood after PE [145].
		Histone modifications	OS influence on histone-modifying enzymes	Changes in histone acetylation in placentas from complicated pregnancies [146].
		microRNA alterations	OS-induced changes in microRNA expression	Altered placental microRNA profiles in PE and IUGR [147].
Mitochondrial Effects		mtDNA mutations	Oxidative damage to mitochondrial DNA	↑ mtDNA mutations in placentas from complicated pregnancies [148].
		Altered mitochondrial dynamics	Changes in fission/fusion balance	Persistent alterations in offspring tissues after IUGR [149].
Vascular and Endothelial Effects		Endothelial progenitor cell dysfunction	Reduced number and function of EPCs	Observed in women with history of PE [150].
		↑ arterial stiffness	Vascular remodeling, endothelial dysfunction	Observed in both mothers and offspring years after preeclamptic pregnancies [151].
Cellular Senescence		Telomere shortening	Oxidative damage to telomeres	Shorter telomeres in placentas from complicated pregnancies [152].
		Senescence-associated secretory phenotype	Persistent low-grade inflammation and pro-oxidant state	↑ senescence markers in preeclamptic placentas [153].

ADHD: Attention Deficit Hyperactivity Disorder; CV: Cardiovascular; DNA: Deoxyribonucleic Acid; EPC: Endothelial Progenitor Cell; IUGR: Intrauterine Growth Restriction; mtDNA: Mitochondrial DNA; OS: Oxidative Stress; PE: Preeclampsia; RNA: Ribonucleic Acid; ↑: Increased/Increase.

**Table 4:** Oxidative stress-associated biomarkers [3,13,27,31,102,154,155,156,157,158,159,160,161,162,163,164,165,166].

Biomarker Category	Examples	Significance in Placental Pathology
Lipid Peroxidation Products [13,154,155]	MDA F2-isoprostanes 4-HNE	Indicate oxidative damage to cellular membranes
Protein Oxidation Markers [156,157,158]	Protein carbonyls AOPPs Nitrotyrosine	Reflect oxidative damage to placental proteins
DNA/RNA Oxidation [159,160,161,162]	8-OHdG 8-hydroxyguanosine	Indicate oxidative damage to nucleic acids
Antioxidant Status [159,161]	SOD, CAT, GPx, Vitamins C and E, Glutathione	Reflect the placenta’s ability to counteract OS
Redox-Sensitive Transcription Factors [163,164]	Nrf2 NF-κB	Indicate changes in gene expression due to OS
Mitochondrial Dysfunction Markers [165,166]	mtDNA copy number Electron transport chain enzyme activities	Reflect OS-induced mitochondrial damage
Gasotransmitters [3,27,31,102]	NO metabolites H_2_S levels CO production	Indicate OS and vascular dysfunction.

MDA: Malondialdehyde; 4-HNE: 4-hydroxynonenal; AOPPs: Advanced oxidation protein products; 8-OHdG: 8-hydroxy-2′-deoxyguanosine; SOD: Superoxide dismutase; CAT: Catalase; GPx: Glutathione peroxidase; OS: Oxidative stress; Nrf2: Nuclear factor erythroid 2-related factor 2; NF-κB: Nuclear factor kappa B; mtDNA: Mitochondrial DNA; NO: Nitric oxide; H_2_S: Hydrogen sulfide; CO: Carbon monoxide.

**Table 5:** Oxidative stress biomarkers in pregnancy complications [167,168,169,170,171,172].

Complication	Role of OS	Key Findings	Potential Biomarkers
Preeclampsia [167]	- Placental ischemia-reperfusion injury - Endothelial dysfunction - Systemic inflammation	- Increased placental OS markers - Reduced antioxidant capacity - Increased circulating anti-angiogenic factors	- sFlt-1/PlGF ratio - Malondialdehyde - 8-isoprostane - Nitrotyrosine
Fetal Growth Restriction [168]	- Impaired placental development - Reduced nutrient transport - Mitochondrial dysfunction	- Increased placental oxidative damage - Altered placental gene expression - Reduced placental antioxidant enzymes	- F2-isoprostanes - Protein carbonyls - 8-OHdG - Mitochondrial DNA damage
Gestational Diabetes Mellitus [169]	- Hyperglycemia-induced ROS production - Mitochondrial dysfunction - Advanced glycation end products	- Increased lipid peroxidation - Reduced antioxidant defenses - Altered placental insulin signaling	- 8-isoprostane - Advanced glycation end products - Reduced glutathione/oxidized glutathione ratio
Preterm Birth [170]	- Inflammation-induced OS - Premature rupture of membranes - Activation of labor pathways	- Increased OS markers in amniotic fluid - Reduced antioxidant capacity in maternal circulation - Oxidative damage to fetal membranes	- F2-isoprostanes in amniotic fluid - Myeloperoxidase - Matrix metalloproteinases
Recurrent Pregnancy Loss [171]	- Impaired trophoblast invasion - Endothelial dysfunction - Oxidative damage to oocytes/embryos	- Increased OS markers in maternal circulation - Reduced antioxidant capacity - Oxidative DNA damage in placental tissues	- 8-OHdG - Lipid hydroperoxides - Total antioxidant capacity
Placental Abruption [172]	- Acute ischemia-reperfusion injury - Activation of inflammatory cascades - Systemic OS in severe cases	- Increased markers of oxidative damage in placental tissue - Elevated inflammatory mediators - Potential alterations in coagulation factors	- Malondialdehyde - Protein carbonyls - Inflammatory cytokines (e.g., IL-6, TNF-α)

DNA: Deoxyribonucleic Acid; sFlt-1: Soluble fms-like tyrosine kinase-1; PlGF: Placental growth factor; 8-OHdG: 8-hydroxy-2′-deoxyguanosine; IL-6: Interleukin-6; TNF-α: Tumor necrosis factor-alpha; ROS: Reactive oxygen species; OS: Oxidative stress.

**Table 6:** Therapeutic approaches targeting oxidative stress (OS) in placental disorders [173,174,175,176,177,178,179,180,181,182,183,184,185,186,187,188,189,190,191,192,193,194].

Approach	Examples	Rationale	Evidence/Status
Antioxidant Supplementation	Vitamins C and E	Free radical scavengers, regenerate other antioxidants	Large RCTs (VIP, DAPIT) showed no benefit in preventing PE. Potential issues with high doses interfering with physiological ROS signaling [173,174].
	Selenium	Essential component of glutathione peroxidase and other selenoproteins	Observational studies show lower selenium in PE. Limited intervention studies with mixed results [175].
	N-acetylcysteine (NAC)	Precursor to glutathione, enhances cellular antioxidant capacity	Small studies show potential benefits in recurrent pregnancy loss and preterm labor. Larger trials needed [176].
	Melatonin	Potent antioxidant that can cross the placenta	Shown to reduce OS and improve outcomes in animal models of IUGR and PE. Early-stage clinical trials ongoing [177].
Targeting Specific ROS Sources	MitoQ, SkQ1 (mitochondria-targeted antioxidants)	Accumulate in mitochondria to reduce mitochondrial OS	Promising results in preclinical models of PE and IUGR. Phase 2 trial of MitoQ in PE completed (results pending) [178].
	Apocynin, VAS2870 (NADPH oxidase inhibitors)	Reduce ROS production from a major enzymatic source	Mostly in preclinical stages. Apocynin shown to improve endothelial function in animal models of PE [179].
	Allopurinol (xanthine oxidase inhibitor)	Reduce ROS production during ischemia-reperfusion events	Some studies in PE and fetal hypoxia. APEX trial ongoing for fetal neuroprotection [180].
Enhancing Endogenous Antioxidant Systems	Sulforaphane, bardoxolone methyl (Nrf2 activators)	Stimulate endogenous antioxidant gene expression	Promising preclinical data, but concerns about potential teratogenicity. Sulforaphane is being studied in gestational diabetes [181].
	SOD mimetics (e.g., tempol), catalase mimetics	Provide enzymatic antioxidant activity without affecting gene expression	Beneficial effects in animal models of PE and IUGR. Human studies limited [182].
Targeting Placental Vasculature and Angiogenesis	L-arginine, sildenafil citrate (NO donors)	Improve placental blood flow and have antioxidant effects	Some positive results in IUGR. STRIDER trials for sildenafil in severe early-onset IUGR (mixed results, safety concerns) [183].
	GYY4137, sodium hydrosulfide (H_2_S donors)	H_2_S has vasodilatory and antioxidant properties	Promising preclinical data in PE and IUGR models. Human studies in early stages [184].
	Pravastatin	Pleiotropic effects including improved endothelial function and reduced OS	Encouraging results in animal models and small human studies. StAmP trial ongoing for prevention of PE [185].
Anti-inflammatory Approaches	Low-dose aspirin	Anti-inflammatory and antiplatelet effects	Recommended for prevention of PE in high-risk women. Meta-analyses show 10–20% risk reduction [186].
	Omega-3 fatty acids	Anti-inflammatory effects and precursors to specialized pro-resolving mediators	Some studies show reduced risk of preterm birth, but effects on PE are inconsistent. ORIP trial showed no benefit for PE prevention [187].
	Targeted anti-cytokine therapies (e.g., TNF-α inhibitors)	Reduce inflammatory signaling that can exacerbate OS	Mostly in preclinical stages for pregnancy complications. Case reports of TNF-α inhibitor use in refractory PE [188].
Modulation of Placental Metabolism	Metformin	Activates AMPK, potentially improving mitochondrial function and reducing OS	Benefits shown in gestational diabetes. EMPOWaR and MOP trials found no benefit for obese pregnant women. Ongoing research in PE prevention [189].
	Dietary interventions (e.g., Mediterranean diet)	Reduce metabolic stress and inflammation	Some observational studies show benefits. ESTEEM trial showed reduced gestational diabetes risk with Mediterranean diet [190].
Novel and Emerging Approaches	Extracellular vesicle-based therapies	Deliver antioxidants or supportive factors directly to the placenta	Early preclinical research. Potential for targeted delivery of therapeutic cargo [191].
	CRISPR-based approaches	Correct genetic factors predisposing to OS or enhance antioxidant gene expression	Theoretical at this stage for placental disorders. Ethical concerns for human application [192].
	Nanomedicine (e.g., nanoparticle-based antioxidant delivery)	Improve targeting and efficacy of antioxidant therapies	Preclinical studies ongoing. Potential for enhanced placental drug delivery [193].
	Gasotransmitter therapies (e.g., inhaled NO, CO-releasing molecules)	Modulate vascular function and reduce OS	Some clinical trials for inhaled NO in preterm IUGR. CO-RMs in preclinical stages [194].

AMPK: AMP-activated protein kinase; CO: Carbon monoxide; CO-RMs: Carbon monoxide-releasing molecules; CRISPR: Clustered Regularly Interspaced Short Palindromic Repeats; IUGR: Fetal growth restriction; H_2_S: Hydrogen sulfide; NADPH: Nicotinamide adenine dinucleotide phosphate; NO: Nitric oxide; Nrf2: Nuclear factor erythroid 2-related factor 2; OS: Oxidative stress; PE: Preeclampsia; RCTs: Randomized controlled trials; ROS: Reactive oxygen species; SOD: Superoxide dismutase; TNF-α: Tumor necrosis factor alpha.

## Data Availability

Data available on request.
